# Current-driven production of vortex-antivortex pairs in planar Josephson junction arrays and phase cracks in long-range order

**DOI:** 10.1038/s41598-018-33467-y

**Published:** 2018-10-18

**Authors:** Francisco Estellés-Duart, Miguel Ortuño, Andrés M. Somoza, Valerii M. Vinokur, Alex Gurevich

**Affiliations:** 1 Universidad de Murcia, Departamento de Física-CIOyN, Murcia, 30071 Spain; 2 Argonne National Laboratory, Materials Science Division, Chicago, IL 60637 USA; 3 Univeristy of Chicago, Computation Institute, Chicago, IL 60637 USA; 4 Old Dominion University, Department of Physics, Norfolk, VA 23529 USA

## Abstract

Proliferation of topological defects like vortices and dislocations plays a key role in the physics of systems with long-range order, particularly, superconductivity and superfluidity in thin films, plasticity of solids, and melting of atomic monolayers. Topological defects are characterized by their topological charge reflecting fundamental symmetries and conservation laws of the system. Conservation of topological charge manifests itself in extreme stability of static topological defects because destruction of a single defect requires overcoming a huge energy barrier proportional to the system size. However, the stability of driven topological defects remains largely unexplored. Here we address this issue and investigate numerically a dynamic instability of moving vortices in planar arrays of Josephson junctions. We show that a single vortex driven by sufficiently strong current becomes unstable and destroys superconductivity by triggering a chain reaction of self-replicating vortex-antivortex pairs forming linear of branching expanding patterns. This process can be described in terms of propagating phase cracks in long-range order with far-reaching implications for dynamic systems of interacting spins and atoms hosting magnetic vortices and dislocations.

## Introduction

Topological defects such as vortices or dislocations determine many key properties of systems with long-range order, including superconductivity, superfluidity, magnetism, liquid crystals, and plasticity of solids^1,2^. These defects are characterized by a topological charge, for example, by an integer winding number *n* in the phase *θ* of the complex order parameter ${\rm{\Psi }}={\rm{\Delta }}\,\exp (in\,\theta )$ for quantized vortices in superconductors. Destruction or creation of a single topological defect in the bulk requires overcoming a macroscopic energy barrier, resulting in the conservation of topological charge. In this work we address the question whether such topologically-protected stability of a vortex can be broken if a rapidly moving, two-dimensional vortex (2D) is driven by a strong force.

The physics of superfast vortices has attracted much attention in light of the development of superconducting qubits and digital memory^3–5^, sources of THz radiation^6^, or radio-frequency superconducting cavities for particle accelerators^7^. Theoretical and experimental investigations have uncovered a wealth of dynamic vortex phases^8–19^, while new imaging tools have made it possible to probe vortices at nanometer scales^20^ and reveal hypersonic vortices moving much faster than the velocity of superfluid condensate^21^. The physics of ultrafast dislocations and their instabilities have also attracted much attention^22–24^. It has been usually assumed that a driven vortex preserves its identity as a topological defect, no matter how fast it moves, because instability of a vortex would violate the conservation of its topological charge. Yet it has been shown recently that a vortex driven by strong current in a long Josephson junction^25,26^ or 1D Josephson junction array (JJA)^27^ can become unstable due to Cherenkov radiation producing a cascade of expanding vortex-antivortex (V-AV) pairs which destroy the global superconducting phase coherence.

The issue of stability of driven topological defects becomes particularly intriguing in 2D systems in which long-range order can be destroyed by the Berezinskii-Kosterlitz-Thouless (BKT) transition resulting from thermally-activated unbinding of V-AV pairs^28,29^ or a superconductor-insulator transition in JJAs^30,31^ which model ultrathin granular films and monolayers^32–34^. So far theories of the BKT transition under current drive have assumed that current only affects thermally-activated or quantum V-AV pair production^31,35^, but the topological defects remain intact. Here we show that long-range order in driven 2D systems can be destroyed by a rapidly moving topological defect which causes a cascade of self-replicating pairs of defects of opposite polarity. For instance, a driven vortex in a planar JJA triggers a cascade of V-AV pairs which destroy the global phase coherence in the way similar to the propagation of cracks or elastic twins is solids^36^.

**Figure 1:**
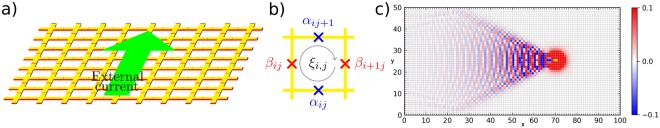
Vortex in a Josephson junction array. (**a**) Cartoon of the array. (**b**) A single plaquette in the array where ${\alpha }_{ij}$ and ${\beta }_{ij}$ are the Josephson phase differences along *x* and *y*, respectively, and $\xi$ the circulation. (**c**) Map of the *z*-component of the magnetic field around a driven vortex calculated at $\eta =0.1$, $\varepsilon =1.0$ and $\gamma =0.575$. The yellow contour line of the circulation represents the vortex core.

We performed numerical simulations of coupled sine-Gordon equations describing a particular case of planar JJAs^30,31^

1 $${\ddot{\alpha }}_{ij}+\eta {\dot{\alpha }}_{ij}+\,\sin \,{\alpha }_{ij}=\varepsilon ({\xi }_{ij}-{\xi }_{ij-1}),$$

2 $${\ddot{\beta }}_{ij}+\eta {\dot{\beta }}_{ij}+\,\sin \,{\beta }_{ij}=\varepsilon ({\xi }_{i-1j}-{\xi }_{ij})+\gamma ,$$

3 $${\xi }_{ij}={\beta }_{ij}+{\alpha }_{ij+1}-{\beta }_{i+1j}-{\alpha }_{ij},$$

where ${\alpha }_{ij}(t)$ and ${\beta }_{ij}(t)$ are phase differences between *i*-th and *j*-th junctions along the *x* and *y* axis, respectively, $\gamma =I/{I}_{c}$, and *I* is a transport current applied uniformly along the *y*-axes, as shown in Fig. 1. Here the dots over ${\alpha }_{ij}(t)$ and ${\beta }_{ij}(t)$ in Eqs (1–3) denote derivatives with respect to the dimensionless time $t{\omega }_{J}$, $\eta =1/{\omega }_{J}RC$ is a damping parameter, *I*_*c*_ is a Josephson critical current, ${\omega }_{J}$ is a Josephson plasma frequency, *R* is a quasiparticle resistance and *C* is a capacitance of a single junction. The parameter $\varepsilon =s{I}_{d}/\xi {I}_{c}$ quantifies inductive coupling of neighboring junctions, where *I*_*d*_ and $\xi$ are the depairing current and the coherence length in superconducting links of length *s* between the junctions. Here we take into account only the kinetic inductance of the superconducting condensate, disregarding long-range charge and inductive coupling between the grains through the electromagnetic stray fields, and focus on classical JJAs with negligible quantum charge effects^30,31^. Generic equations (1–3) can model dynamics of driven topological defects in many systems with long-range order, including artificial JJAs and granular superconducting films^30,31^, magnetic vortices and domain walls described by a classical XY model^37^, commensurate-incommensurate transitions and domain walls in charge density waves^38–41^, or dislocations in crystals^36^. The 2D systems are special as they exhibit a continuous BKT transition due to unbinding of topological defects in equilibrium^28,29^.

## Results

We addressed the behavior of driven topological defects by simulating Eqs (1–3) for a 50 × 100 array with periodic boundary conditions along *y* and open boundary conditions along *x* at the edges of the array. Vortices were revealed by the circulation matrix ${\xi }_{ij}$ which identifies the vortex core in a cell where the sum of circulations along the plaquette is close to 2*π*. Because ${\xi }_{ij}$ only contains the circulation of the phase differences on the Josephson junctions but does not include phase gradients in the superconducting links due to circulating current in the vortex, ${\xi }_{ij}$ does not vanish outside the plaquette where the core is centered but decreases with the distance from that plaquette. In the subsequent figures the core boundary is outlined by the contour along which the sum of ${\xi }_{ij}$ equals *π*. To visualize the field of a moving vortex, we also calculated the *z*-component of the magnetic field ${H}_{z}(x,y,z)$ at the hight *z* above the array using the discrete Biot-Savart law:

4 $${H}_{z}(x,y,z)={H}_{0}\,\sum _{x^{\prime} y^{\prime} }\,\frac{(y-y^{\prime} ){J}_{x}(x^{\prime} ,y^{\prime} )-(x-x^{\prime} ){J}_{y}(x^{\prime} ,y^{\prime} )}{|{(x-x^{\prime} )}^{2}+{(y-y^{\prime} )}^{2}+{z}^{2}{|}^{3/2}},$$

where $${H}_{0}={\mu }_{0}{J}_{c}/4\pi {s}^{2}$$, all lengths are in units of *s*, $${J}_{x}(x^{\prime} ,y^{\prime} )={\ddot{\alpha }}_{ij}+\eta {\dot{\alpha }}_{ij}+\,\sin \,{\alpha }_{ij}$$ and $${J}_{y}(x^{\prime} ,y^{\prime} )=$$$${\ddot{\beta }}_{ij}+\eta {\dot{\beta }}_{ij}+\,\sin \,{\beta }_{ij}$$ are the components of currents at position *x*′, *y*′ at the midpoint of each junction. We used a moving frame that keeps the vortex in the middle of the array after the vortex was initially introduced as explained in the Methods. The simulations were continued until a stationary state was reached, and the vortex velocity was obtained from the updating rate of frames.

### Steady state behavior

Our calculations show that at small currents $\gamma  < {\gamma }_{p}$ the vortex is immobilized by intrinsic pinning in a JJA, in agreement with previous works^30,31^. If *γ* exceeds the depinning current *γ*_*p*_, the vortex moves and emits Cherenkov radiation along with bremsstrahlung caused by hopping of a vortex in the discrete JJA. Cherenkov radiation with the wave vectors **k** appears if the velocity of the vortex is smaller than the Swihart velocity at $k=0$ but exceeds the phase velocity ${v}_{\phi }({k}_{x},{k}_{y})$ of small-amplitude waves in the JJA at finite *k*_*x*_ and *k*_*y*_, where ${v}_{\phi }({k}_{x},{k}_{y})$ decreases as *k*_*x*_ and *k*_*y*_ increase^25,30,31^. For instance, Fig. 1 shows a snapshot of the field map $h(x,y)={H}_{z}(x,y,z)/{H}_{0}$ in a moving vortex calculated from Eqs (1–3) at $z=s$. The radiated wake in the Cherenkov cone behind the vortex and the interference of waves reflected from the edges are apparent, the radiation appears at currents well below *I*_*c*_. The amplitude of Cherenkov wake diminishes as the damping parameter $\eta$ increases, and radiation behind moving vortices was detected at $\eta$ up to 0.5. As the inductance parameter $\varepsilon$ increases radiation becomes more intense.

**Figure 2:**
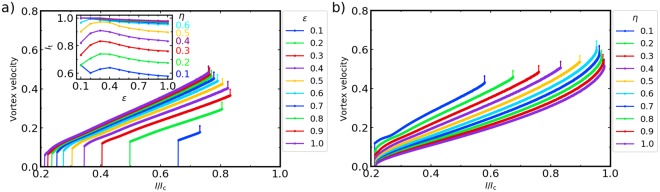
Vortex velocity. (**a**) Vortex velocity as a function of current *γ* for different values of $\varepsilon$ and $\eta =0.3$. Each curve starts at the depinning current ${\gamma }_{p}(\varepsilon )$ and ends at the threshold current *γ*_*s*_, marked with a vertical arrow indicating the transition to a dynamic state with no long range order. Inset shows the threshold current ${\gamma }_{s}(\varepsilon )$ as a function of $\varepsilon$ for several values of $\eta$. (**b**) Vortex velocity as a function of current $\gamma$ for different values of $\eta$ and $\varepsilon =0.3$.

The stationary velocity of the vortex $v(\eta ,\varepsilon ,\gamma )$ was calculated as a function of current *γ* for underdamped arrays with $\eta  < 1$ for which the effect of radiation is most pronounced. In our simulations the driving current $\gamma =kt$ was gradually increased with a small ramp rate $k=5\cdot {10}^{-6}$ to avoid artifacts due to transient instabilities. Shown in Fig. 2a is a mean vortex velocity *v*(*γ*) as a function of current calculated for $\eta =0.3$ and different values of $\varepsilon$. Likewise, Fig. 2b shows *v*(*γ*) calculated for $\varepsilon =0.3$ and different values of $\eta$. In both cases $v=0$ below the depinning current ${\gamma }_{p}(\varepsilon )$. The velocity has a jump at $\gamma ={\gamma }_{p}$ and then increases smoothly up to a second critical current *γ*_*s*_ at which the second jump in *v*(*γ*) occurs.

In the region of currents ${\gamma }_{p} < \gamma  < {\gamma }_{s}$ the vortex moves with a steady-state velocity controlled by the balance between the driving Lorentz force and the drag forces caused by ohmic currents in the junctions and radiation losses, as illustrated by Fig. 1c. Here the threshold current *γ*_*s*_ calculated for different values of $\eta$ is a nonmonotonic function of the inductance parameter $\varepsilon$, as shown in the inset in Fig. 2. The velocity of the vortex *v*(*γ*) defines the dc electric field-current characteristics of the array in the flux flow state, $E(I)=v{\varphi }_{0}{n}_{v}$, where *n*_*n*_ is the areal density of vortices and ${\varphi }_{0}$ is the magnetic flux quantum.

### Types of instabilities

**Figure 3:**
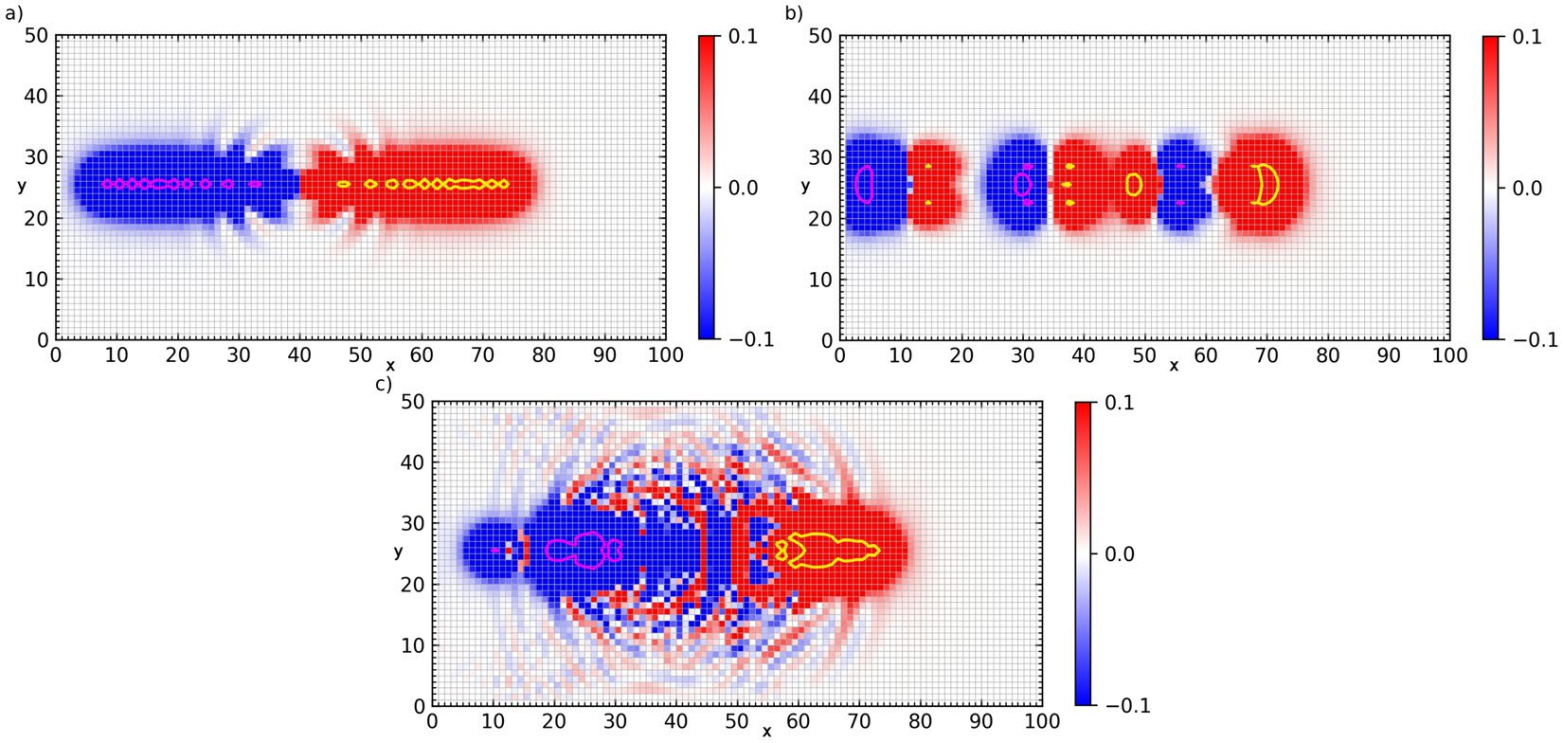
Vortex/antivortex pair production. Color maps the *z*-component of the magnetic field together with contour maps of the current circulation, showing the vortex core. (**a**) Line V-AV pair production along the direction of motion calculated at $\eta =0.5$, $\varepsilon =1.0$ and $\gamma =0.898$. 2D waves radiated from the central zone of V-AV pair production are apparent. (**b**) Transverse branching of the topological dipole expanding along the *x*-axis in an underdamped array calculated at $\eta =0.1$, $\varepsilon =1.0$ and $\gamma =0.579$. (**c**) Splitting instability for the higher dissipation where vortex/antivortex pairs are generated at the upper and lower tips of the initial vortex ($\eta =1.0$, $\varepsilon =1.0$ and $I=0.99$). In this case the vortices structure grows in the vertical direction and the radiation wake is not visible.

**Figure 4:**
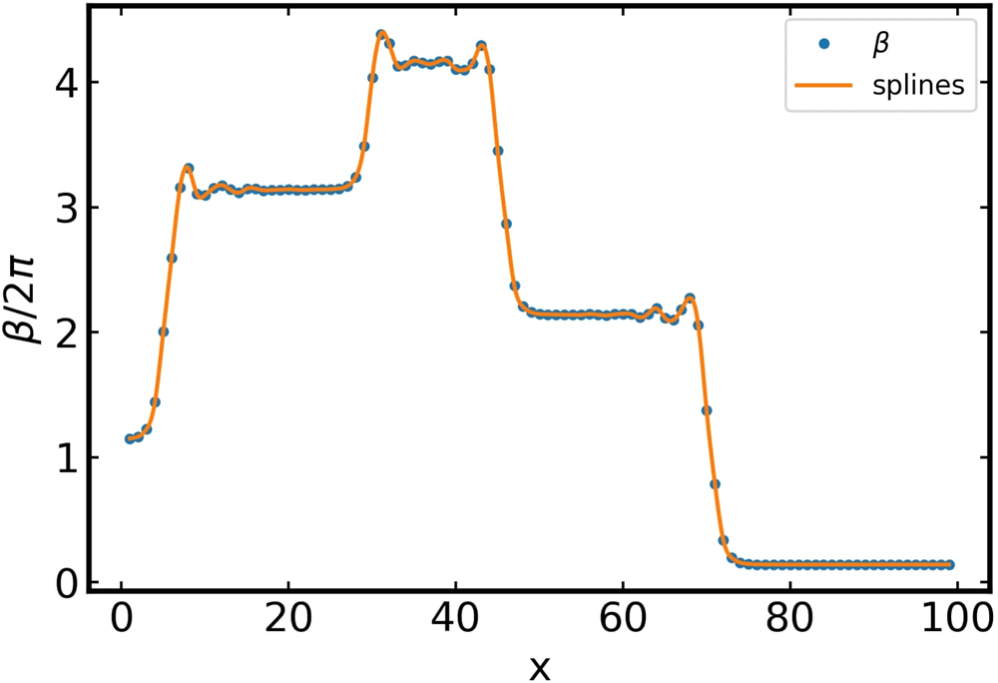
Phase difference profile. A snapshot of the phase difference ${\beta }_{x,L/2}$ on the row of junctions along the initial V-AV pair propagation. Each ascending step in *β*_*x*_ corresponds to an antivortex and the decending step to a vortex, so that the net phase difference 2*π* between the left and right ends remains fixed. Calculation was done for $\eta =0.3$, $\varepsilon =1.0$ and $\gamma =0.775$.

As *γ* exceeds *γ*_*s*_ the steady-state vortex becomes unstable, causing striking dynamic patterns shown in Fig. 3. The instability proliferates from the Cherenkov wake behind a moving vortex where a critical nucleus is formed by the junctions being in the unstable state with the phase differences confined between *π*/2 and 3*π*/2, similar to that of a driven vortex in a long JJ^25,26^. Manifestations of this instability are different for weak $(\eta \ll 1)$ and moderate $(\eta \gtrsim 1)$ damping, yet in any case the dynamic structures represented in Fig. 3 result from continuous production of V-AV pairs and the subsequent separation of vortices and antivortices which accumulate at the opposite sides of an expanding domain. The pattern shown in Fig. 3 can be regarded as an expanding dipole of growing positive and negative topological charges with a fixed net topological charge of the initial vortex. This process bears a remarkable similarity with the crack propagation in crystalline solids where the relevant topological defects are edge dislocations^36^. Indeed, a chain of vortices and antivortices produces a staircase phase difference ${\rm{\Delta }}\beta (x)$ on the Josephson junctions along the chain as shown in Fig. 4. For the spatially separated V and AV cores marked by the yellow and magenta contours in Fig. 3a, the phase jumps on the junctions connecting the aligned V-AV pairs first increase with the distance from the last vortex, reaches the maximum in the middle of the chain and then decreases to zero at the other end of the chain^25^. As the vortex pattern expands, the staircase distribution of phase differences along the V-AV chain evolves into a growing dome-like ${\rm{\Delta }}\beta (x,t)$ which can be regarded as a “phase crack” in the long-range superconducting order.

In underdamped arrays represented by Fig. 3a,b the expanding vortex dipole is mostly caused by Cherenkov and bremsstrahlung radiation. At $\gamma  > {\gamma }_{s}$ the amplitude of the radiation wake behind the moving vortex exceeds a threshold of generation a V-AV pair. Here the critical wake first produces an expanding V-AV pair which in turn generates enough radiation to produce two more V-AV pairs, triggering a subsequent V-AV chain reaction and continuous pair production. Our simulations performed in a wide range of $\varepsilon$ and $\eta$ have shown that in a moderately underdamped array $(\eta \lesssim 1)$ this radiation mechanism produces V-AV pairs aligned along the direction of motion of the initial vortex. Despite a rather complex 2D magnetic field pattern shown in Fig. 3a, the multiquantum vortex dipole essentially results from a 1D Cherenkov instability similar to that was observed in previous simulations of the Cherenkov vortex instability of vortices in nonlocal Josephson junctions^25,26^ or 1D junction arrays and dislocations in the Frenkel-Kontorova model^27^. As $\eta$ increases, the amplitude of radiation wake decreases, so the instability occurs at higher currents *γ*_*s*_ which are still smaller than the critical current of the Josephson junctions $(\gamma =1)$.

Shown in Fig. 3a are the color map of the magnetic field and the circulations ${\xi }_{ij}$ at the initial stage of the V-AV pair production, where the red and blue regions correspond to vortices and antivortices, respectively, and the yellow and purple contours outline the boundaries of vortex cores. Figure 3a is characteristic of V-AV pairs production in a moderately underdamped array with $\eta =0.5$ where the V-AV pairs propagate along the trajectory of the initial vortex. One can see waves radiated from the central zone where the V-AV pairs are generated. As the damping parameter $\eta$ decreases, radiation becomes more pronounced and the vortex structure branches in the transversal direction, as shown in Fig. 3b.

At $\eta \gtrsim 1$, dissipation suppresses the radiation vortex instability but another mechanism of V-AV pair production takes over at currents close to the critical current *I*_*c*_ of the Josephson junctions, ${\gamma }_{s}\to 1$. In this case shown in Fig. 3c V-AV pairs are formed two at a time on both sides of the original vortex. Then two V-AV pairs move apart in such a way that two vortices coalesce with the initial vortex into a multi-quantum vortex and two antivortices move in the opposite direction. Next, two more V-AV pair form at the tips of the macrovortex and the process repeats, resulting in a growing chain of multiquanta vortices and antivortices, each of them keep generating doublets of V-AV pairs at their tips. This regime is characteristic of moderately overdamped arrays with $0.5\lesssim \eta \lesssim 1$, for which our simulations show practically no apparent manifestations of Cherenkov radiation. Movies of dynamic phase patterns at different parameters obtained in our simulations are available at Supplementary Material.

### Phase diagram

**Figure 5:**
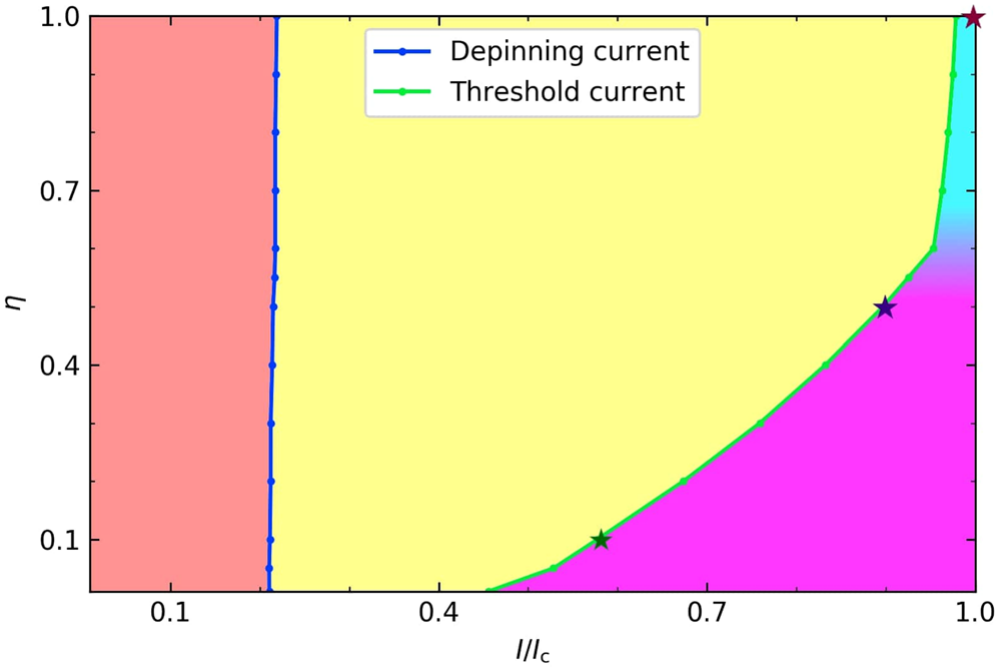
Phase diagram. The $\eta -\gamma$ phase diagram was calculated from the data shown in Fig. 2b at $\varepsilon =1$. Here the read region corresponds to a pinned vortex and the yellow region corresponds to a stable moving vortex. The stars show the values of the parameters for which the color maps in Fig. 3 were calculated.

The results presented above are summarized in a $\eta -\gamma$ phase diagram shown in Fig. 5. The phase diagrams calculated for different values of $\varepsilon$ turned out to be qualitatively similar, so we show here a representative case of $\varepsilon =1$. Here the red region $\gamma  < {\gamma }_{p}(\varepsilon )$ corresponds to the pinned vortex, and the yellow region corresponds to a stable vortex moving with a constant mean velocity (see Fig. 1). The uniform motion of the vortex is unstable below the green line $\eta ={\eta }_{s}(\gamma ,\varepsilon )$. In turn, the unstable region is divided into two regions corresponding to two different mechanisms of generation of V-AV pairs represented in Fig. 3: the magenta region corresponds to the radiation-assisted generation of V-AV pairs, and the cyan region corresponds to the doublet V-AV pair emission. Here the color maps shown in Fig. 3a,c were calculated for the parameters corresponding to the two stars in Fig. 5. The transition between the line and doublet V-AV pair production occurs in a crossover region where the slope of the green boundary line ${\eta }_{s}(\gamma )$ increases sharply. As the parameter $\varepsilon$ decreases, the depinning and threshold lines shift to the right.

## Discussion

Our simulations of JJAs revealed rich dynamics of driven vortices controlled by interplay of ohmic losses, Cherenkov radiation and bremsstrahlung. Emission of V-AV pairs by a moving vortex at $\gamma  > {\gamma }_{s}$ can be described in terms of propagating phase cracks in the order parameter, which can be applicable to driven topological defects in a wide class of systems, including vortices in JJAs, granular superconducting films or superfluid films, magnetic vortices and domain walls or dislocations in crystals. Dynamic states of propagating phase cracks can be controlled by current and the damping parameter, giving rise to either 1D self-replication of V-AV pairs and branching phase cracks at $\eta \ll 1$ or generation of doublets of V-AV pairs in the transverse direction producing beads of macrovortices at $\eta \simeq 1$, as illustrated in Fig. 3.

The above radiation mechanism of vortex splitting resulting in the subsequent V-AV pair production in the 2D JJAs is markedly different from the well-known proliferation of pairs of Abrikosov vortices and antivortices from the edges of a wide, current-carrying superconducting film where a transition of a chain of expanding Abrikosov V-AV pairs into a phase slip can occur^21,42,43^. The Cherenkov V-AV pair production triggered by a moving vortex in single long JJ in wide thin film or monolayers^25,26^ can be strongly affected by pinned Abrikosov vortices in the electrodes. Interaction of Josephson and Abrikosov vortices^44,45^ could in principle be used to tune the V-AV pair production in long Josephson junctions. In large planar junctions, the V-AV pair production results in expanding Josephson vortex loops^46^.

In conclusion, this work addresses a fundamental question of what happens to long-range order if a driven topological defect becomes unstable. We show that the long-range order in driven 2D systems can be destroyed by a single topological defect triggering a cascade of self-replicating pairs of defects of opposite polarity. This process can be initiated be either a quenched defect which is already present in the system (for example, a trapped vortex in a superconductor or a residual dislocation in a crystal) or appears due to thermal fluctuations. The resulting V-AV pair production can manifest itself in jumps on the V-I characteristics in underdamped granular superconductors at $I\ll {I}_{c}$, the jumps being unrelated to Joule heating^47^. Moreover, one can expect new effects of current on the vortex-charge duality and superconducting-insulator transition in Josephson arrays and granular films^30,31^. Propagation of phase cracks could be one more mechanism of jumps on V-I characteristics observed in granular thin films at the onset of insulator transition^48,49^ which so far have been attributed to electron overheating^50,51^. The V-AV pair production triggered by a moving vortex can greatly enhance the output signal in single photon detectors based on arrays of Josephson junctions^52,53^. Our results also suggest a mechanism of initial stage of origination and propagation of mechanical cracks or twins initiated by dislocations in graphene and other atomic monolayers under strong tensile stress.

## Methods

The 4th order Runge-Kutta method has been used to solve the set of equations (1–3) numerically. The implementation of the boundary conditions has been imposed through the circulations (${\xi }_{ij}$). For periodic boundary conditions, the circulation of each plaquette at the top boundary of the array has been forced to be the same as the circulation at the corresponding plaquette at the bottom boundary, and vice versa. For open boundary conditions, all the circulations at the left and right boundaries have been set equal to zero.

Unlike the XY model, where there is a freedom of choosing the phase of an element, in our JJA model we deal with phase differences between the junctions and it is not trivial to establish a vortex configuration. In order to do so, we construct an initial environment that includes a weak link in the direction of the current which triggers the appearance of vortices. At position $i=25$ and $j=0$, we include a factor $\chi$ in the sine term:

5 $${\ddot{\beta }}_{ij}+\eta {\dot{\beta }}_{ij}+\chi \,\sin \,{\beta }_{ij}=\varepsilon ({\xi }_{i-1j}-{\xi }_{ij})+\gamma $$

The value of $\chi =0.01$ was chosen in our simulations. If the current *γ* is high enough then a cascade of equispaced vortices starts to emerge from the weak link. The distance between vortices (or the generation rate) depends on the current, and we choose a value low enough to produce a clear isolated vortex. Once the vortex is far from the weak link, the configuration of *α*s and *β*s is stored in order to be used as the initial configuration of successive runs.

To avoid the vortex reaching the right end of our system, we used a moving frame. The full configuration of the array is shifted by a single plaquette when the center of the vortex reaches a certain position (equal to the lattice spacing). The updating rate gives us the vortex velocity.

The way the velocity at Fig. 2 is calculated has some limitations if we have more than one vortex. Frames are updated according to the position of the maximum circulation plaquette of the whole array, which may not corresponds to the original vortex after the first V-AV emission, i.e., for $\gamma  > {\gamma }_{s}$. Furthermore, when new vortices appear they reach the left boundary in a short time with the corresponding leak of topological charge.

## Electronic supplementary material

n5e10I898

n10e10I99

n1e10I579
